# Mindfulness-based cognitive therapy in obsessive-compulsive disorder: protocol of a randomized controlled trial

**DOI:** 10.1186/s12888-014-0314-8

**Published:** 2014-11-18

**Authors:** Anne Katrin Külz, Sarah Landmann, Barbara Cludius, Birgit Hottenrott, Nina Rose, Thomas Heidenreich, Elisabeth Hertenstein, Ulrich Voderholzer, Steffen Moritz

**Affiliations:** Clinic of Psychiatry and Psychotherapy, University Medical Center Freiburg, Freiburg, Germany; Department of Psychiatry and Psychotherapy, University Medical Center Hamburg-Eppendorf, Hamburg, Germany; Esslingen University of Applied Sciences, Esslingen, Germany; Schoen Clinic Roseneck, Prien, Germany

**Keywords:** Mindfulness-based cognitive therapy, Obsessive-compulsive disorder, Randomized controlled trial

## Abstract

**Background:**

Obsessive-compulsive disorder (OCD) is a very disabling condition with a chronic course, if left untreated. Though cognitive behavioral treatment (CBT) with or without selective serotonin reuptake inhibitors (SSRI) is the method of choice, up to one third of individuals with obsessive-compulsive disorder (OCD) do not respond to treatment in terms of at least 35% improvement of symptoms. Mindfulness based cognitive therapy (MBCT) is an 8-week group program that could help OCD patients with no or only partial response to CBT to reduce OC symptoms and develop a helpful attitude towards obsessions and compulsive urges.

**Methods/design:**

This study is a prospective, bicentric, assessor-blinded, randomized, actively-controlled clinical trial. 128 patients with primary diagnosis of OCD according to DSM-IV and no or only partial response to CBT will be recruited from in- and outpatient services as well as online forums and the media. Patients will be randomized to either an MBCT intervention group or to a psycho-educative coaching group (OCD-EP) as an active control condition. All participants will undergo eight weekly sessions with a length of 120 minutes each of a structured group program. We hypothesize that MBCT will be superior to OCD-EP in reducing obsessive-compulsive symptoms as measured by the Yale-Brown-Obsessive-Compulsive Scale (Y-BOCS) following the intervention and at 6- and 12-months-follow-up. Secondary outcome measures include depressive symptoms, quality of life, metacognitive beliefs, self-compassion, mindful awareness and approach-avoidance tendencies as measured by an approach avoidance task.

**Discussion:**

The results of this study will elucidate the benefits of MBCT for OCD patients who did not sufficiently benefit from CBT. To our knowledge, this is the first randomized controlled study assessing the effects of MBCT on symptom severity and associated parameters in OCD.

**Trial registration:**

German Clinical Trials Register DRKS00004525. Registered 19 March 2013.

## Background

Obsessive Compulsive Disorder (OCD) is a very disabling condition and the fourth most common mental illness with a lifetime prevalence of 1-3% and a 12-month-prevalence of 1-2% [1]. Individuals with OCD experience recurrent intrusive thoughts or impulses (obsessions) and/or repetitive behaviors (compulsions). If left untreated, obsessive-compulsive disorder usually takes a relapsing course and becomes chronic [2,3], associated with substantial impairment of quality of life [4,5] and limitations in social and professional life [6]. Thus, the disorder causes high costs, not only for the affected individuals but also for their families, the health system and society (see [7] for a review).

Cognitive Behavioral Therapy (CBT) with Exposure and Response Prevention (ERP) and/or pharmacological treatment with Selective Serotonin Reuptake Inhibitors (SSRI) is the evidence-based treatment of choice for patients with OCD [8,9]. For CBT with ERP, high pre-post-effect-sizes of roughly Cohen’s *d* = 1.5 and controlled effect sizes around 1.1 are reported [10]. The effectiveness of CBT with ERP is yet limited by substantial rates of patients who do not or only partially respond, suffer relapses or discontinue therapy. About thirty-five percent of the patients do not respond to the treatment recommended by current guidelines in terms of a reduction of symptom severity of at least 35% [11–13]. In addition, the probability of relapse over a 2-year period after achieving remission has been found to be 48% [14]. Especially for the subgroups of patients who do not satisfactorily benefit from CBT with ERP, complementary or alternative treatment strategies must be developed.

Mindfulness-Based Cognitive Therapy (MBCT) [15] is a manualized intervention program in an outpatient group format consisting of eight sessions. It combines mindfulness exercises with elements of cognitive therapy and has originally been designed to prevent relapse in depression. As has been shown in randomized controlled trials of two independent research groups, MBCT as an add-on to treatment as usual (TAU) reduces the probability of relapse in patients with three or more previous depressive episodes when compared to TAU alone [16–18]. A recently published study [19], however, found that MBCT reduced risk of relapse compared with treatment as usual only for participants who had experienced childhood trauma. In a study using an uncontrolled design, MBCT proved to be helpful in patients with acute depressive symptoms: Significant symptom reductions with large effect sizes have been observed after the intervention [20]. In contrast, the findings of a small randomized controlled study on MBCT in seasonal affective disorder suggest that MBCT is not effective in the prevention of disorder recurrence [21]. Several research groups have adapted the original manual for patients with other psychiatric disorders. According to current reviews, medium effect sizes for MBCT in various disorders have been found [22,23]. In randomized pilot studies, positive effects of MBCT have been demonstrated in patients with panic disorder and generalized anxiety disorder [24] and social phobia [25]. In patients with primary insomnia, symptom reductions have been observed after a treatment combining mindfulness with elements of cognitive-behavioral interventions in uncontrolled studies [26,27]. So far, however, no randomized controlled study exists concerning the application of MBCT in patients with OCD. This seems amazing, since training of paying attention to the present moment in an open, friendly and non-judgemental way as cultivated through mindfulness practice might be a powerful way for OCD patients to deal with their intrusive, unwanted and often shaming thoughts and urges [28].

In line with this, several intervention studies hint at the efficacy of mindfulness-skills in reducing OC-symptoms. In a controlled study on a student sample, subclinical OC symptoms were observed to be significantly reduced after a mindfulness training [29]. In the study by Kim and colleagues [24], in patients with generalized anxiety disorder or panic disorder, a significant decrease on the OC-scale of the Symptom Checklist-90-Revised (SCL-90-R), a self-rating instrument assessing global psychopathology, was observed. However, in this study, OC-symptoms have not been assessed with more specific instruments targeting OCD [24]. Some case studies also hint that mindfulness might be useful for patients with OCD (e.g. [30]). In a recent review, Bluett et al. [31] found that acceptance and commitment therapy (ACT), a therapeutic approach focusing on mindfulness techniques and values, was equally effective as manualized treatments such as cognitive behavioral therapy in OCD spectrum disorders.

To our best knowledge, manualized mindfulness interventions like MBCT and MBSR have not yet been investigated in controlled trials in patients with OCD at all. Besides, there are no data on the long term effects of mindfulness interventions in patients with OCD up to now, though mindfulness might be a valuable adjunct to CBT [32].

Based on these considerations, our workgroup recently conducted an open pilot study on Mindfulness Based Cognitive Therapy for patients with Obsessive Compulsive Disorder [33,34]. The intervention consisted in an 8-session MBCT program adapted to OCD [35] which closely followed the original manual by Segal, Williams, and Teasdale [15]. Qualitative and quantitative methods were used, in order to both reflect the subjective experiences of the participants with the treatment program and, on the other hand, to examine objectifiable changes in clinical symptoms. Twelve patients with residual OC symptoms after CBT completed the program and were examined immediately after the intervention and 6 months later. The results showed that MBCT was feasible in patients with acute OCD and was well accepted by the participants. From baseline to post assessment, a significant reduction of the OC symptoms (p = .008) was observed for the total scale of the Y-BOCS and the compulsions subscale (p = .004) with effect sizes within the medium range. Results remained stable at follow-up. We will therefore conduct a bicentric trial on a large sample to examine, for the first time, the effectiveness of MBCT compared to a psychoeducative program (OCD-EP) in OCD patients in order to control for unspecific group effects such as validation of experiences and mutual assistance.

## Methods/Design

### Study design

**Figure 1:**
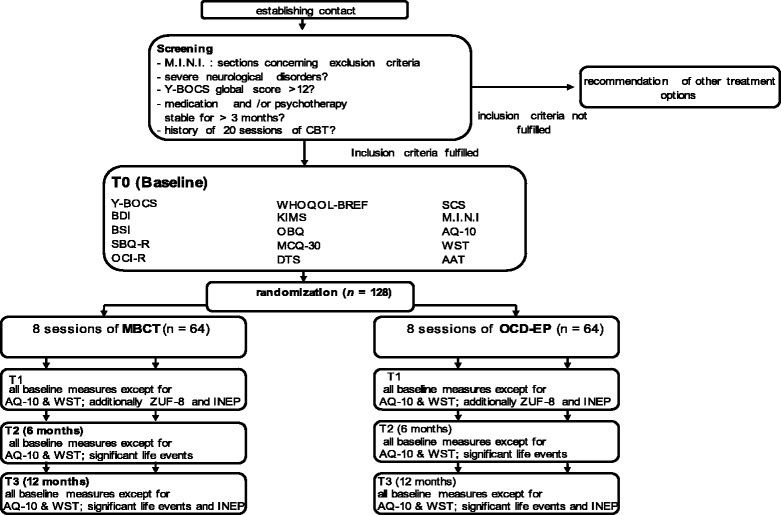
**Study design and measurement time points (CONSORT chart).** AQ-10 = autism spectrum quotient short version; AAT = approach avoidance task; BDI = Beck Depression Inventory II; BSI = Brief Symptom Inventory; KIMS = Kentucky Inventory of Mindfulness Skills; MBCT = Mindfulness Based Cognitive Therapy; MCQ-30 = Metacognitions Questionnaire with 30 Items; WST = Test of Word Power; OBQ = Obsessive Beliefs Questionnaire; OCD-EP = Obsessive Compulsive Disorder Education Program; OCI-R = Obsessive-Compulsive Inventory; SBQ-R = Suicidal Behaviors Questionnaire-Revised; M.I.N.I Mini-International Neuropsychiatric Interview; INEP = Inventory of negative effects of psychotherapy; WHOQOL-BREF = World Health Organization Quality of Life-BREF; Y-BOCS = Yale Brown Obsessive Compulsive Scale.

The study design is shown in Figure 1. In a prospective, bicentric, assessor-blinded, randomized, actively-controlled clinical trial, 128 patients with OCD according to DSM-IV-criteria will be randomly assigned to one of two groups: the studied intervention consists of 8 two-hour-sessions of MBCT, the control condition consists of 8 two-hour-sessions of OCD-EP. MBCT and OCD-EP will be comparable regarding treatment setting.

In the proposed trial, we hypothesize that in patients with OCD who are still symptomatic (i.e., Y-BOCS >12 points) after CBT, MBCT produces a significant reduction of OC symptoms from baseline to post assessment compared to an OCD education program (OCD-EP). A cutoff-score of 12 points is an established criterion of remission proposed by Simpson et al. [11].

The long-term outcomes of MBCT and OCD-EP six and twelve months after the interventions will be compared in an explorative manner. Means, the rates of treatment responders, the rates of patients who achieved remission and the rates of patients who relapsed will be compared. Treatment response and remission will be defined according to the criteria by Jacobson and Truax [36]. Their method includes the calculation of a) a cutoff for the symptom severity after the treatment, and b) an index for a reliable symptom improvement (reliable change index, RCI). Patients who fulfill both criteria will be classified as remitted. Patients who fulfill criterion b) will be classified as responders, even if their post-treatment score is still above the cutoff. Relapse is defined as a return to pre-treatment severity as recommended by Simpson et al. [37]. Patients who were classified as remitted after the intervention will be eligible for the analysis of relapse rates [38].

Further exploratory research questions include the effects of MBCT on secondary outcome measures such as comorbid depressive symptoms (as measured by the Beck Depression Inventory), Quality of Life (World Health Organization Quality of Life BREF), the general psychopathology (Brief Symptom Inventory), the tendency to be mindful in everyday life (Kentucky Inventory of Mindfulness Skills), distress tolerance (Distress Tolerance Scale), self-compassion (Self-Compassion Scale) and dysfunctional beliefs and attitudes (Obsessive Beliefs Questionnaire, Metacognitions Questionnaire). The relationship between mindfulness, distress tolerance and dysfunctional attitudes on the one hand and changes of clinical symptoms on the other hand will be addressed in exploratory analyses. Possible negative effects of the MBCT and OCD-EP treatment will also be explored. Additionally the change in avoidance tendencies will be assessed using an approach avoidance task (AAT) [39] measuring avoidance tendencies to contamination and checking related words and pictures. A subgroup of the sample will also take part in an additional experience-sampling task to examine changes in positive and negative affect, distress caused by OC symptoms, insight into the unreasonableness of obsessions as well as acceptance of unpleasant feelings before and after treatment. The experience sampling method (ESM) [40] is characterised by the repeated collection of real-time data on participants’ momentary inner states in their typical environments, thereby aiming to minimize memory biases and ensuring ecological validity [41].

### The interventions

Both interventions will consist of eight sessions with a length of 120 minutes each, which will be held at weekly intervals in an outpatient setting at the University Medical Center of Freiburg and the University Medical Center Hamburg. Each treatment group will consist of eight to twelve participants.

MBCT is based on mindfulness training as developed by Jon Kabat-Zinn at the University Medical Center of Massachusetts, combined with psychoeducational elements from cognitive therapy. A detailed description of the original intervention can be found in Segal, Williams & Teasdale [15]. In the proposed trial, a modified version of the manual will be applied: The mindfulness elements are adopted from the original manual, and the cognitive elements are tailored to patients with OCD [35].

We intend to use an education program as an active control condition in order to control for unspecific effects of a group treatment. The program (OCD-EP) consists of a presentation and education about the etiology, mechanisms and maintaining factors of OC symptoms, metacognitive and neurobiological perspectives on OCD, information on existing pharmacotherapeutic and psychological treatment modalities and relapse prevention, group sharing and discussion. Behavior therapy techniques or mindfulness interventions are not part of the program.

The treatment sessions will be held by experienced behavioral therapists with longstanding experience in MBCT respective psychoeducation of OCD.

### Setting

Overall, eight MBCT- and eight OCD-EP-groups will be conducted (four MBCT- und four OCD-EP-groups at each site). The baseline assessments (T0) and post-assessments (T1) will be performed directly before respectively after the interventions. The first follow-up (T2) will be six months and the second (T3) will be 12 months after the end of the intervention.

The two participating centers will be the University Medical Center Freiburg and the University Medical Center Hamburg-Eppendorf. Both centers will recruit equal proportions of participants. Potential distortions due to the bicentric approach will be controlled by a stratification and by homogeneous study conditions (same room equipment, same training of the therapists, same measures, etc.).

The bicentric study was approved by the ethics commission of Freiburg University Medical Center. In accordance with the Declaration of Helsinki, all participants will be informed in detail about the design and purpose of the study. Each participant will be required to sign a written consent form and will have the freedom to withdraw consent and quit the study at any point in time. Data will be documented anonymously in a case report file (CRF), which will be stored at the University Medical Center Freiburg for 10 years. All necessary personal data will be kept confidential. All sessions will be videotaped, supervised and checked for adherence. Supervision will be provided by Prof. Thomas Heidenreich, University of Esslingen.

### Inclusion and exclusion criteria

The main inclusion criterion of our study is a primary diagnosis of obsessive-compulsive disorder (ICD-10 F42.-). Participants should suffer from clinically relevant OC symptoms, i.e. a Y-BOCS global score >12 or subscore of ≥8 in either obsessions or compulsions at study inclusion. Participants are required to be between 18 and 70 years of age and to give informed consent. Since we aim at testing the efficacy of MBCT for patients who are still symptomatic after treatment, only patients who completed at least 20 sessions of CBT with ERP prior to study inclusion will be eligible.

The criteria are aiming at a preferably wide generalizability of the results. However, patients who require a different treatment due to comorbid conditions or whose condition gives reason to expect an inability to benefit from a group treatment will be excluded from participation. These are patients with current manic episode or manic episode within 5 years before study inclusion (ICD-10 F30.-), patients with severe depressive episodes according to ICD-10 criteria, a history of psychosis (ICD-10 F20-F29), current substance dependence (ICD-10 F1x.2), borderline personality disorder or Asperger syndrome (ICD-10 F84.5). Further exclusion criteria are suicidal tendencies according to the Suicidal Behaviors Questionnaire-Revised (SBQ-R), severe neurological disorders or an intelligence quotient <70 as measured by the Test of Word Power (WST). Moreover, to prevent confounding effects, patients with begun or altered psychotherapeutic treatment or medication within 12 weeks before study inclusion are also excluded. A change of dosage within the same drug, however, is tolerated.

### Sample size

A power analysis was performed with the program “G*Power” [42]. Based on the pilot data, on results of comparable studies [24,43] and on theoretical considerations, we assume a between-group effect size of *d* = 0.5 favoring MBCT. Assuming an a priori test power of 0.8, a total sample size of *n* = 128 (*n* = 64 per intervention) will be sufficient to detect this effect by an *F*-test in an ANOVA at *p* < .05 level of significance for a group × timepoint interaction. We assume a dropout rate of approx. 15% and thus target a total sample size of *n* = 148.

### Outcome measures

#### Primary outcome

The Yale Brown Obsessive Compulsive Scale (Y-BOCS) is a semi-structured clinical interview which is considered the gold standard for measuring OCD severity [44,45]. The Y-BOCS will be used as primary outcome in the proposed trial.

#### Secondary outcomes

The Beck Depression Inventory-II (BDI) is a common self-rating assessing the severity of depressive symptoms. The instrument features good characteristics for internal consistency, test-retest reliability and validity [46]. The subjective quality of life will be measured by the World Health Organization Quality of Life–abbreviated (WHOQOL-BREF), an interculturally valid, multidimensional questionnaire with good to excellent psychometric properties [47]. The 18-item version of the Brief Symptom Inventory (BSI) is a short, reliable and valid self-assessment instrument for general psychopathology [48]. The 20-item-version of the Kentucky Inventory of Mindfulness Skills (KIMS) will be used to measure dispositional mindfulness [49]. The instrument has good discriminant and convergent validity and is sensitive to changes over the course of MBCT [50]. The Obsessive-Compulsive Inventory Revised (OCI-R) is a self-rating scale assessing the major symptoms of OCD on six dimensions (see [51] for the German version). The Obsessive Beliefs Questionnaire (OBQ) is a multidimensional instrument assessing dysfunctional metacognitive beliefs in the context of OCD. The questionnaire has shown a satisfactory reliability and validity (see [52] for the German version) and is sensitive to therapeutic changes [53]. In addition to the OBQ, the short form of the Metacognitions Questionnaire (MCQ-30) will be used. The MCQ-30 is a brief questionnaire with good indices for reliability and validity [54]. The instrument’s sensitivity to therapeutic changes has been shown for the long version [55]. The Distress Tolerance Scale (DTS) [56] measures the capacity to experience and withstand negative psychological states. As a German version of the instrument is not yet available, the DTS was translated by our workgroup. Moreover, the self- compassion scale (SCS) measures the capability of cultivating self-kindness, common humanity and mindfulness even when things go bad instead of self-judgment, feelings of isolation and over-identification (see [57] for the German version).

Diagnoses will be made by trained staff using the Mini International Neuropsychiatric Interview (M.I.N.I.). The M.I.N.I. is a structured diagnostic interview for DSM-IV and ICD-10 psychiatric disorders [58]. The Suicidal Behaviors Questionnaire-Revised (SBQ-R) [59] and the Test of Word Power (WST), which is a short, valid instrument assessing general intelligence [60] will be administered at baseline. Besides, the Autism Spectrum Quotient (AQ) [61] will be applied at baseline for assessment of autistic traits. In addition, sociodemographic variables and a questionnaire concerning satisfaction with the result of randomization and motivation, constructed by the workgroup will be administered. The medication status will be enquired and documented at all times of assessment. Subjective treatment satisfaction will be assessed after the program by the Treatment Satisfaction Questionnaire (ZUF-8). Moreover, a questionnaire called “Side-Effects of Psychotherapy” scale (SEPS), based on items of the Inventory of Negative Effects of Psychotherapy (INEP) [62], a scale assessing negative effects of psychotherapy in different areas of life, as well as items constructed by the workgroup assessing negative outcomes of the intervention, will be administered post treatment and follow-up. At both follow-ups, participants will be asked about significant life events within the follow-up period.

In addition, an approach-avoidance-task (AAT) [39], a computerized neuropsychological paradigm, will be applied by comparing response times for OCD-related (checking and contamination) and neutral words and pictures, before and after treatment. In this task, patients are instructed to respond to each stimulus presented on the computer screen by pushing or pulling a joystick. When participants push the joystick away from themselves, the picture on the screen shrinks, creating the visual impression that the stimulus moves away. When they pull the joystick, the picture grows until it fills the screen, creating the impression of an approaching stimulus. Response times for pulling and pushing stimuli away can be compared in order to assess automatic avoidance tendencies. A similar version of the AAT used in the present study has already been applied in another study on OCD patients [63].

Moreover, in order to investigate the participants’ experiences in situ as an additional outcome measure of MBCT and to gain further insight into the relationship of emotions and OCD symptoms, the Experience Sampling Method (ESM) [40] will be applied to a subset of the study sample before and after treatment. Participants will be asked to record their OC symptoms, the distress caused by symptoms, affective states and acceptance of unpleasant affective states respectively on an electronic device 6 times a day on 6 consecutive days before and after the group interventions. The prompts to fill out a form will be given at random time-points between 8 am and 22 pm by the web-based experience sampling software *movisensXS* (movisens GmbH, Karlsruhe). Partici\pants who decide to take part in this additional paradigm will receive 50€ for compliance rates of at least 80% and a short summary of their mood profile and symptoms before and after treatment.

### Statistical analyses

Between-group comparisons at baseline will be performed using analyses of variance for the continuous variables and Fisher’s exact test for the categorical variables. Means and standard deviations will be calculated for descriptive purposes. The main analyses will be computed using analyses of covariance in view of statistical studies suggesting that controlling for the baseline score is superior to simple pre-post comparisons and usually leads to an increase in power. An advantage of this type of analysis over mixed models is that it accounts for baseline differences and regression to the mean (i.e. higher scores usually yield greater improvement). The rates of patients achieving response and/or remission and the relapse rates in both groups will be compared using Fisher’s exact test. The time to relapse will be estimated using Kaplan-Maier survival analyses. Survival curves will be compared using a log-rank-test. A cox-regression will be performed to analyze variables influencing the time to relapse. In order to prevent distortions, the intent-to-treat principle will be used. Multiple imputation will be adopted to estimate post-treatment and follow-up scores for non-completers (i.e. no data available at reassessment). The multiple imputation principle is considered superior to the last observation carried forward method (LOCF) [64]. When using the LOCF, follow-up data can be difficult to interpret [10].

## Discussion

To the best of our knowledge, this is the first study to evaluate the efficacy of MBCT in patients with OCD as a complement to CBT with ERP. Based on the results of the pilot study outlined above, the proposed project aims at systematically examining the impact of MBCT as a new treatment option for patients with OCD who do not sufficiently benefit from CBT with ERP, who suffer a relapse or who experience difficulties in partaking in this kind of treatment.

We also intend to undertake a wide range of additional analyses. Various factors have been discussed that could, individually or in their combination, mediate positive effects of mindfulness training in patients with OCD. One of them is an increased willingness to experience difficult thoughts, feelings and body sensations [32]. A study on patients with Social Phobia provided evidence that Mindfulness Based Stress Reduction, a therapy program that is closely linked to MBCT, improves emotion regulation skills in patients with social phobia [65]. This could be especially relevant for patients with OCD, because emotion regulation has been identified as a common function of OC symptoms [66]. We therefore will examine changes of distress tolerance and their association with therapeutic outcome. Moreover, the mindfulness approach might be helpful to question metacognitive styles and beliefs that maintain OCD such as thought-action fusion (TAF) [67,68] and thought control [69]. Thus, we will use measures of dysfunctional metacognitions and believes in explanatory analysis in order to identify factors that mediate or moderate therapeutic effects. Besides, we will evaluate the impact of self-compassion on changes of symptom severity. Self-compassion has shown to be below average in OCD patients [70] and was a better predictor of symptom severity and quality of life than mindfulness in patients with depression and anxiety disorder [71]. Especially for patients with OCD, self-compassion could be helpful to disengage from struggling with shaming obsessions such as violent or sexual intrusions and to deal with them in an accepting, not self-judging manner. Furthermore, subgroup analyses on the influence of baseline characteristics, such as baseline severity, sociodemographic variables, comorbid mental disorders and treatment motivation are planned.

We will further examine approach avoidance tendencies as measured by the AAT, because patients struggle to change their obsessions and compulsions even though they have insight into the irrationality of their fears. According to 2-systems models, (e.g. [72]) behavior is influenced by an automatic, inflexible impulsive system and a slower and more flexible reflective system. In OCD it is assumed that in the impulsive system, strong connections between obsessive related concepts and items are associated with danger [73]. Therefore the AAT offers us a chance to detect changes in behavioral tendencies that patients may not be able to report.

There is a growing body of empirical evidence showing that mindfulness-based interventions are effective in increasing positive and/or in decreasing negative affect in a variety of clinical conditions [74–77]. Since there is no study exploring changes in positive and negative affect caused by MBCT in OCD patients so far, we decided to use the ESM paradigm in order to fill this gap and to provide further insight into the nature of OC symptoms and associations with affective states as they occur in the natural environments of participants.

Regarding recruitment of participants, we decided to not exclude patients who currently undergo psychopharmacological or psychotherapeutic treatment. One reason for the liberal acceptance of concomitant treatments refers to practical considerations. We assume to reduce the non-eligibility rate and to improve external validity, since the vast majority of patients with clinically relevant OCD receive some kind of treatment. Another reason is the chance to conduct subgroup analyses in order to evaluate the efficacy of MBCT in patients with OCD as a complement to current treatment. However, the acceptance of concomitant treatment may also constitute a limitation of the study, since the power to detect a difference between MBCT and OCD-EP might be reduced due to effects of other treatment modalities.

Finally, we hope that the 6-month and the 12-month follow-up data will provide important insights in longterm-effects of mindfulness-based interventions in OCD on severity of symptoms and related measures such as obsessional beliefs or quality of life.

In view of the large percentage of OCD patients who do not sufficiently benefit from conventional CBT with stimulus exposure and response prevention, there is a strong urge to find additional or complementary treatment options. The present study wants to shed light on the role of MBCT as a valuable intervention to boost the gains from cognitive-behavioral approaches in OCD by opening up new avenues of relating to OC symptoms.
